# Governance of Assisted Living in Singapore: Lessons for Aging Countries

**DOI:** 10.3389/fpubh.2022.868246

**Published:** 2022-06-14

**Authors:** Si Ying Tan, Luting Poh, Jeremy Lim

**Affiliations:** ^1^Leadership Institute for Global Health Transformation, Saw Swee Hock School of Public Health, National University of Singapore, Singapore, Singapore; ^2^Department of Pharmacology, Memory Aging and Cognition Centre, Yong Loo Lin School of Medicine, National University of Singapore, Singapore, Singapore

**Keywords:** assisted living, age-friendly environment, long-term care, case study, governance, regulation

## Abstract

A global trend toward aging populations means that the challenge of providing adequate long-term care to older people looms large in many countries. In Singapore, a public discourse revolving around the expansion of assisted living to create age-friendly environments in long-term care has emerged. This study examines Singapore's experience in developing regulations for assisted living by documenting the different levels of regulation in place and by identifying the regulatory gaps remaining to govern assisted living. Anchoring in a conceptual framework on the governance of assisted living, different regulatory components of assisted living at the micro-, meso-, and macro-levels are analyzed. Using a case study method, primary and secondary data examining the experiences of governing and implementing assisted living in Singapore were collected. Analysis was conducted using a thematic analysis approach. Micro- and some macro-level regulations, which include admission assessment, staffing, and infrastructural requirements for assisted living, are maturing and evolving, while meso-level regulations, such as operational management, the monitoring framework, and stipulations for training requirements for staff, remain a work-in-progress in Singapore. The regulations for assisted living are currently primarily guided by soft laws, such as practice guidelines; the government has committed toward enacting permanent regulations for all long-term care facilities with the phased implementation of the Health Care Services Act from 2021 to 2023. We conclude that assisted living, despite the early stage of its development in Singapore, is a viable care model that should be expanded to meet the rising demand for care on the part of a majority of older people, who fall in the middle of the care continuum (that is, they can neither live independently nor need complete institutionalization). We also propose five policy recommendations for all aging countries to strengthen the governance of assisted living in long-term care. These include establishing (i) clear provisions on care quality assessment and the redress of grievance, (ii) minimum standards of care, (iii) differential regulations for assisted living, (iv) routine care assessment, and, (v) applying technology in assisted living facilities to address a shortage of care workers.

## Introduction

Population aging is touted as a success story of development as improved socio-economic conditions bring forth better healthcare, nutrition, and quality of life. The world's population has been aging fast for three decades. In 1990, there were 703 million people aged 65 and above; by 2019, this number had doubled. In a similar vein, the share of the population aged 65 and above has increased from 6% in 1990 to 9% in 2019. Likewise, the oldest of the elderly population (aged 80 and above) also tripled between 1990 and 2019, and this trend is projected to persist until 2050, with the effects observed most significantly in Asia and North Africa. Correspondingly, life expectancy has also increased substantially, in line with socio-economic improvement. As of today, life expectancy at birth for females and males is 74.7 and 69.9, years, respectively, and it is expected to continue increasing till 2050 (1).

**Figure 1:**
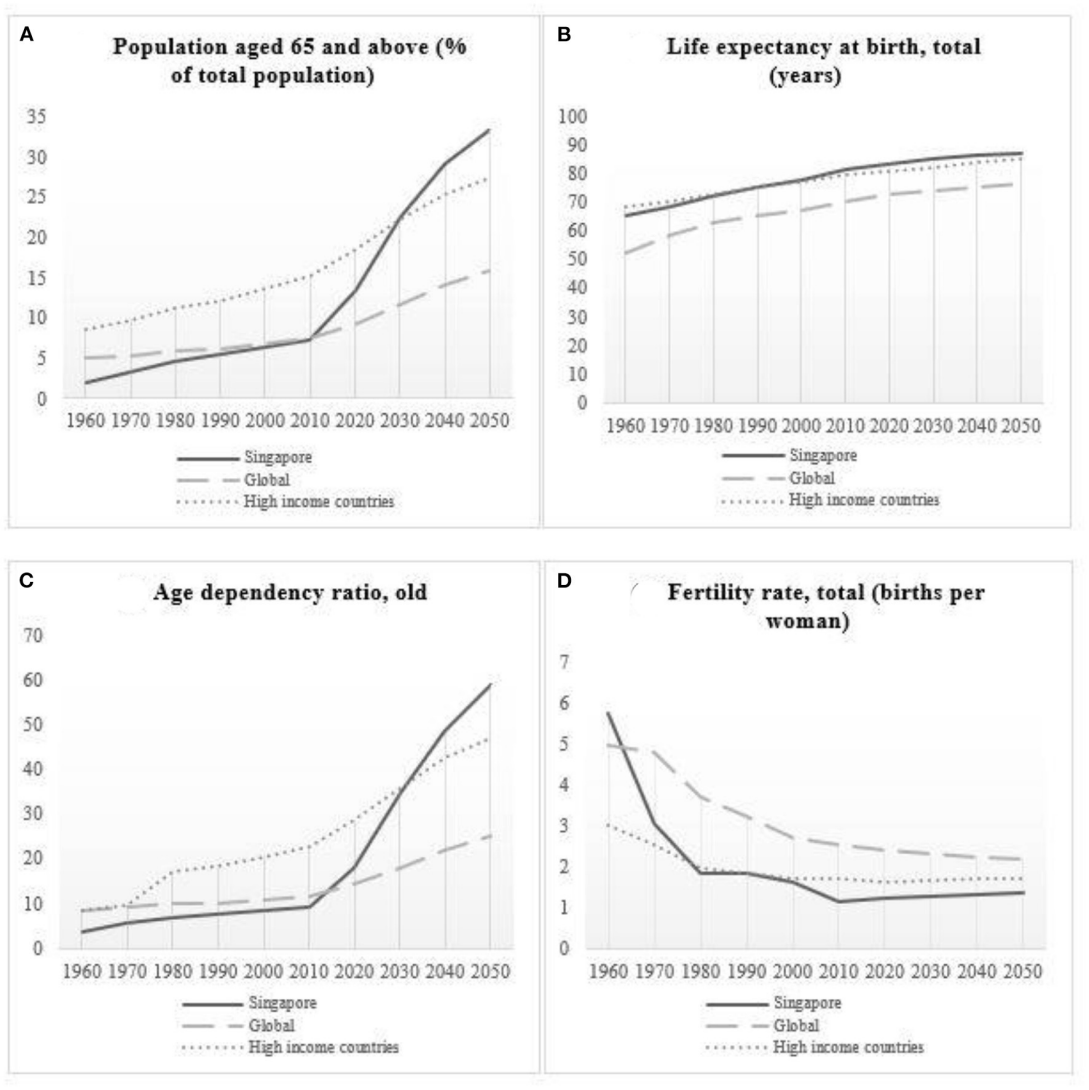
**(A)** Trends in the population aged 65 and above (% of total population); **(B)** Life expectancy at birth, total (years); **(C)** Age dependency ratio, old; **(D)** Fertility rate, total (births per woman) in Singapore, the world, and high-income countries from 1960 to 2050.

As an industrialized and rapidly aging nation, Singapore too is grappling with the immediate and long-term implications of a graying population. In 2020, older people aged 65 and above stood at approximately 14% of the total population (1 in 7) (2). By 2050, it is forecasted that the proportion of older people aged 65 and above will be approximately 33.3% (1 in 3) (3). Singapore residents have also consistently ranked among the highest in the world in terms of life expectancy at birth. In 2019, the life expectancy at birth for the resident population was estimated at around 83.6 years (3). Furthermore, the old-age dependency ratio in Singapore, which is currently among the highest in the world, increased from 3.7 in 1960 to 18.0 in 1970, and is projected to reach 58.5 by 2050 (The World Bank, 2021). Compounding the aging population issue in Singapore is the steep fall in the total fertility rate, which decreased drastically, from 5.8 in 1960 to 1.2 in 2020 (4). Figures 1A–D illustrate the rising and falling trends, the projections of population aged 65 and above, life expectancy at birth, the old-age dependency ratio, and the total fertility rate in Singapore, comparing these with the global average and the average from the high-income countries between 1960 and 2050.

The rapid aging of the population in Singapore will translate into higher demand for long-term care services over the next few decades. Assisted living is an emerging long-term care option in Singapore, owing both to a graying population and the policy narratives that it is a desirable model of old-age living and could address the care needs of most senior citizens (5). North American countries possess a relatively longer history in growing the assisted living market, as compared to other parts of the world. Between the late 1970s and the early 1980s, there was a paradigm shift in the long-term care sector in the US, from traditional nursing homes to assisted living; this was prompted by a reconsideration of nursing homes as the predominant service delivery model in long-term care, one largely motivated by the variation in their quality (6). The aim of assisted living is to enable residents to exercise autonomy, privacy, and independence in their daily lives, besides preserving dignity and respect in a nurturing environment that is non-restrictive and home-like (7). It is also intended to preserve the physical, mental, and psychological health of individuals who seek to lead independent lives, despite needing a certain degree of assistance (8). With inclusivity in its overall policies and sufficient human resources, assisted living can enable aging-in-place by creating an age-friendly environment that is conducive for retirement (9). In aging country such as Japan, living in an age-friendly environment was shown to promote social participation and civic engagement among the older people, while allowing them to maintain their social networks (10).

A systematic review of the regulations for assisted living across the world demonstrated heterogeneities in the implementation models, even within the same jurisdiction. It also identified a knowledge gap in the understanding and design of regulations of assisted living beyond North America (11). To fill this gap, this study aims to explore the introduction and expansion of assisted living in the long-term care sector in Singapore by examining the development of regulations that govern two types of assisted living facilities (ALFs): public and private. We posed the following questions. What are the different levels of regulation that are already in place to govern ALFs in Singapore? What are the remaining regulatory gaps, and how can insights into these inform policy and practice in other aging countries?

**Figure 2:**
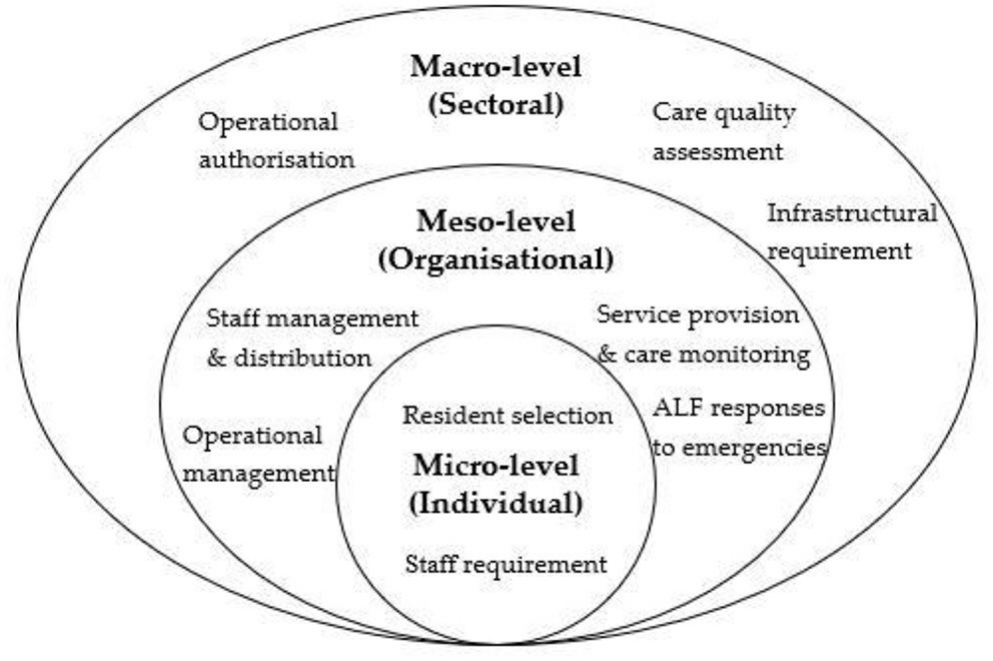
Assisted living governance framework.

We applied a three-level governance of assisted living framework that specifies macro-, meso-, and micro-level regulatory components for assisted living developed by a systematic review (11). The theoretical underpinnings of this framework were borrowed from the American public administration literature and motivated by the same regulatory set-up in the administration of public agencies (11). Micro-level regulations are apparatuses that apply to individual actors involved in the care setting, and the components of regulations include staff requirements and resident selection (assessment criteria). In terms of the meso-level regulations, the authority can impose rules on the ALFs as operating units. It is also possible for the service providers to construct their own operational frameworks, based on the state's requirements and guidelines. This level of regulation entails components such as staff management and distribution, service provision and care monitoring, operational management, and ALF responses to emergencies (such as the Covid-19 pandemic). At the macro-level, regulations are intended to achieve sectoral-wide uniformities to define the implementation architecture of the industry. Regulatory components at this level entail operational authorization, quality assessment, and infrastructural requirements (11) (see Figure 2).

## Materials and Methods

An in-depth analysis examining the governance and implementation experiences of assisted living in long-term care in Singapore was conducted using a case study approach from January to March 2021. The case study approach is a widely applied method for understanding and theorizing circumstantial events; it centers on the examination of a contextualized contemporary phenomenon by dissecting its real-life context (12). Intended to be “an intensive study of a single unit for the purpose of understanding a larger class of similar units” [(13), 342], the case study has the potential to derive unique insights from studying a geographical phenomenon and infer similar observations to other comparable jurisdictions.

To build the case narratives, information was drawn from various sources such as journal articles, local and international news articles, public agency websites, published and unpublished policy documents, reports from social service organizations, and law and statutory documents. The incorporation of various information sources enabled the triangulation of data for verification and cross-validation to achieve credibility and trustworthiness in data collection (13). We built the case study by, first, identifying and extrapolating the emerging themes in our data, thus anchoring the conceptual framework. These emerging themes were later delineated and strengthened using the thematic analysis approach, through line-by-line reading and coding of our interview notes and secondary data files (14). During this process, we also incorporated explanation-building and pattern-matching techniques of the case study method to ensure consistency of the accounts and the information within the themes (12).

## Results

### Case Description: The Expansion of Assisted Living as a Model for Long-Term Care in Singapore

Situated in the middle of the spectrum of long-term care services—between aging at home and aging in institutions such as nursing homes—ALFs aim to provide a comprehensive range of long-term care services to older people who are physically and mentally functioning to a certain extent, and would like to access social care without being admitted to a nursing home. Assisted living espouses the core concepts of healthy aging, social care, and connection to the community. These core concepts are supplemented with the ethos of assisting older people to conduct most of the activities in daily lives themselves by encouraging normalcy, promoting a certain level of independence, and preserving dignity and personal autonomy. Through promoting normalization and encouraging mutual support among the residents, assisted living aims to delay institutionalization and future-proof the care needs of older people (15).

In Singapore, ALF took root slightly earlier in the private sector than in the public sector. The first private ALF started operation in Singapore in 2015, whereas the public assisted living units known as the Community Care Apartments were launched by the Housing Development Board (HDB) in December 2020. Three tenancy models—the hospitality model, the hybrid model, and the home model—were proposed for the private ALF sphere. The hospitality model provides higher-end services that incorporate lifestyle and leisure services; the hybrid model provides residential, basic social care services, and some add-on non-essential services; the home model provides residential and basic social care services (16). The hospitality and hybrid models can be designed as independent living at purpose-built or refurnished facilities such as condominiums, while the home model is best operated in refurbished private homes (16). The public ALFs were constructed in the form of public housing flats known as Community Care Apartments. The first public ALFs, known as the pilot Community Care Apartments, were launched in Bukit Batok in February 2021 for seniors aged 65 and above. A total of 160 apartment units were launched under the Build-To-Order exercise (a public housing scheme that allows buyers the flexibility of selecting the location and type of flat desired and knowing upfront when their down-payment is expected) by the HDB and will be fully built to house senior residents from 2024 onwards (17).

Different funding modes have been proposed for the public and private ALFs in Singapore. Operated and constructed by the Ministry of Health Holdings Vanguard Health, the public ALFs are currently sold as leases to prospective residents who are at least 65 years old with an average gross monthly income lower than S$14,000. Prospective tenants are given the flexibility to purchase leases ranging from 15 to 35 years (in 5-year increments), as long as the leases cover the applicants and their spouses up to the age of 95. The prices of these leases, which include the flat price and a basic service package throughout the period of the lease, range from S$67,000 to 107,000. This sum of money is partially refunded if the residents do not live through their whole original lease period or if they return the flat to HDB (17). The purchase of the ALF leases is fundamentally based on the assumption that older people can liquidate their existing housing assets, whether public or private, to purchase these leases, which can see them through the “silver years” of their lives.

In the private sphere, the proposed funding models are somewhat different. While the mainstream model is the monthly rental model, there has been a proposal to implement two other types of funding model: the membership fee structure model and the reverse mortgage model. The monthly rental payment model, priced at approximately S$3,500 per month, includes a monthly rent, monthly care fees (with different tiers of care depending on the care needs of individuals, based on the nurse's assessment), and other consumable items. The membership fee structure model encompasses an upfront early purchase price, which is a confirmative freehold or leasehold payment for a unit prior to moving in; this purchase signifies their “membership.” Subsequently, the member would need to pay for the services through a monthly service fee. Finally, the deferred management fee is payable only when the resident leaves the community. The reverse mortgage model is a flexible loan model which allows residents to post their properties as collaterals while living in their properties. No loan repayment is required for this model till the homeowner passes away, sells the property, or moves out. Individuals could fund their care in private ALFs either through their personal savings, contributions from the family, public and/or private annuities, or through the liquidation of their existing housing assets (16).

### Case Analysis

#### The Development of Micro-Level Regulations

There have been separate criteria developed for resident selection and staff requirements for public and private ALFs.

Residents in public and private ALFs are admitted based on specific assessment criteria. In the public ALFs, the applicants for the Community Care Apartments are able to opt for priority or non-priority schemes. Preference is given to older people who need more assistance in activities of daily living (ADL) under the priority schemes. The current functional status assessment framework (for other publicly funded community care assistance schemes) is applied for applicants (18). Essentially, there is a combination of older people who possess different levels of functional capabilities if they are assessed to be fit for living in the Community Care Apartments (18). In the private ALFs, a policy guide has been developed to admit residents who require partial ADL or who require instrumental activities of daily living (IADL). Apart from the standard admission criteria, they are assessed for hearing, vision, and cognitive function, based on a minimal mental state examination. Regular health assessments (at least once a year) have also been proposed for the private ALF residents (16).

In terms of staff requirement, the public ALFs or the Community Care Apartments have on-site community managers who assist the residents by linking up the necessary services and resources according to their needs, organizing community activities to engage the residents, and managing simple household tasks for the residents. There are also additional care staff who work in shifts. They monitor the safety of the residents on a 24-h basis and conduct social programmes for the residents. These staff are hired by the future operators of the Community Care Apartments. As for the private ALFs, it is proposed that the core team of the care staff should comprise a caregiver manager, caregivers, and other specialized staff, such as nurses, medical social workers, therapists, dietitians, cooks, and transport staff, who cater to the specific needs of the residents (17).

#### The Development of Meso-Level Regulations

In terms of operational management, similar approaches have been proposed for both public and private ALFs. For public ALFs, the on-site community manager and care staffs are the main respondents to the residents' needs. When anomalies are detected, the management team flag these to the various professionals in the health and long-term care services contracted by the operators. In the private ALFs, the roles stipulated for the caregiver managers is akin to the community managers in the public ALFs, albeit with slightly more comprehensive responsibilities. These responsibilities include, are but not limited to, managing the admission of the residents, preparing the caregiver roster, ensuring infection control, providing emergency response, overseeing all activities of the residents, managing volunteers, and supervising the caregiver team (16).

In terms of service provisions, the public ALFs offer a basic service package and additional services that can be supplemented based on the residents' needs. The basic service package entails arranging for a wide range of care and support services, simple home fixes, communal activities at the community spaces, basic health checks, 24-h emergency response, and key card access to individual flats (17). As for the private ALFs, the services, depending on the fee structures, typically include basic care and health services (24-h basic and first-aid care, on-demand care services when residents activate them, assistance with ADLs and IADLs when necessary, medication management, and regular health checks and monitoring), dining services, and other social activities and programmes, such as fitness exercises, food outings, hobby activities, and pastoral care (16).

To ensure ALF staff are competent to manage the residents' needs, it has been proposed that the ideal staff member–resident ratio is between 1:8 and 1:10. In addition, initial and specific training to care for residents with special care needs, such as cognitive impairment, hearing impairment, visual impairment, mobility issues, and mental health issues, has been proposed as prerequisites for the care staff in private ALFs. The modules for initial training should include areas such as: (i) managing the expectations of residents in terms of the services provided; (ii) reporting requirements; (iii) general infection control and policies for the residents; (iv) emergency preparedness; and (v) preventing the potential abuse, neglect, or exploitation of the residents. Even though no minimum educational qualification is required, it is stated that all care staff in private ALFs should be suitably qualified, preferably with specific training in dementia and ALF management skills; they should also have accreditation with the appropriate government agencies (16). As of January 2022, the stipulations for training requirements for staff and staff–resident ratios in public ALFs are still being developed.

In addition to the standard service provision, it is also important for ALFs to protect their residents during emergency situations. This is especially critical during disease outbreaks as residents in the long-term care setting are highly vulnerable to infections due to their declining health and the communal living, which necessitates space-sharing. In general, all the long-term care providers in Singapore are overseen by the Ministry of Health and the Agency for Integrated Care. At the beginning of the Covid-19 outbreak in 2020, these two organizations outlined a set of safe management strategies for nursing homes, day-care centers, and ALFs in Singapore. Several key precautionary measures were implemented: (i) the suspension of large-scale senior-centric gatherings; (ii) strict adherence to the personal protective equipment usage guidelines for staff, volunteers, visitors, and seniors; (iii) mandated entry approvals for all returning foreign care workers; (iv) enhanced safe distancing guidelines through split zones and movement control; and (v) the provision by operators of alternate housing for on-site staff displaying Covid-19 symptoms (19).

#### The Development of Macro-Level Regulations

In terms of infrastructure design, the public ALFs have specific designs which enable older people to live independently. They include constructing senior-friendly fittings, such as grab bars, wheelchair-accessible bathrooms, slip-resistant flooring, built-in wardrobes, cabinets, furnished kitchens, and a sliding door that enables the residents to partition their living room and bedroom spaces based on their preferences. Aside from individual units, a communal space is included in each floor for the residents to establish social connections and build their social networks. In addition, basic amenities, such as retail, leisure, healthcare, and public transport, are within reach. Underneath these Community Care Apartments, there are other amenities that have been designed to promote assisted living, such as a precinct pavilion, a strolling path, a fitness station, a community garden, and a hawker centre (17).

The private ALFs are also designed to enable older people to have their designated private space and a common space where they can mingle with other residents. The private space includes their bedrooms, which are furnished with senior-friendly fittings and furniture, and non-slip surfaces for in-suite bathrooms. The common spaces include kitchen and dining amenities that allow them to dine either independently or with assistance, and other common areas that enable them to engage with various indoor and outdoor activities. Ideally, the surrounding public spaces, such as public parks, supermarkets, grocery stalls, food centers, and salons, should also be close to the ALFs and be easily accessible (16).

In terms of infrastructure capacity, land scarcity remains an ongoing issue for Singapore. In long-term care provisions, the government continues to grapple with this issue when confronted with questions pertaining to replicating public ALFs in most neighborhoods and communities in the future. It is important that the construction of these ALFs should be close to other health and other long-term care facilities to facilitate care transitions. Furthermore, to preserve the spirit of aging-in-place without having to move older people out from their familiar communities, public ALFs will have to be constructed effectively and sustainably in the face of competing developmental priorities for the allocation of public land.

A comprehensive instrument for care quality assessment in ALF has yet to be developed. While it is envisioned that ALF will serve the care needs of a large segment of seniors who are partially independent, assisted living could potentially be stretched across a wider continuum of long-term care services to include care for individuals in the end stage of their lives as well.

Since September 2021, Singapore has phased in the implementation of the Health Care Services Act as a legislative measure to broaden the regulatory scope for the existing Health Care Services Bill. The purpose is to introduce services-based licensing for different health services (including long-term care), strengthen quality assurance, and step up the safeguards for residential care services (20). For instance, a new requirement for Service Review Committee will be instituted for selected services and programmes that are deemed higher risk, more complex, and of public interest to govern the quality of all services falling under the purview of the Health Care Services Act (21) (see Table 1 for the summary of the development of micro-, meso- and macro-level regulations of ALF in Singapore).

**Table 1:** Regulatory development of assisted living in Singapore.

**Regulatory components**	**Public ALFs**	**Private ALFs**
**Micro-level regulations**		
Resident selection and assessment	• Comprise priority and non-priority schemes. • Priority schemes will give preference to those who need more ADL assistance. Assessment based on the prevailing functional assessment method for other home and community care services.	• Residents will undergo ADL and IADL assessment, hearing, vision and cognitive functions assessment, and regular health assessment (usually done annually) as screening measures to determine their eligibility.
Staff requirement	• General: On-site community managers, other care staff. • Specific (on demand): Specific staff will be contracted by the operators.	• General: Caregiver managers and other caregivers. • Specific (on-demand): Nurses, medical social workers, therapists, dietitians, cooks, transport staff.
**Meso-level regulations**		
Operational management	• On-site community manager and care staffs are the main respondents to the residents' needs. • The management team will flag any anomalies identified to the various professionals in the health and long-term care services contracted.	• The managers oversee the admissions and daily activities of the residents, preparing caregiver roster, infection control, emergency response, managing volunteers, and supervising the caregiver team.
Service provision and care monitoring	• Basic service package (care and support services, home fixes, everyday activities, basic health checks, 24-h emergency response) and additional ala carte services (laundry, rehabilitation, nursing, medical and other social care services). • Monitoring framework is currently in policy discussion.	• Basic care and health services (24 h basic and first-aid care), medication management, ADL/IADL assistance, regular health checks, on-demand care services, dining services, social activities, food outings, hobbyist activities, pastoral care. • Monitoring framework has not been developed.
Staff management and distribution	• Stipulation of trainings required for staff work-in-progress. • Recommended staff-resident ratio work-in-progress. • No minimum educational qualification for staff.	• Initial and specific trainings have been proposed as requisites for the care staff in ALFs. • Recommended staff-resident ratio 1:8 to 1:10. • No minimum educational qualification for staff.
Responses to emergencies	• Emergency response guidelines prescribed by the Ministry of Health (MOH) and the Agency for Integrated Care (AIC) for all long-term care facilities during the Covid-19 pandemic. • For example, key precautionary measures were implemented under the advisories issued by the above two organizations.	
**Macro-level regulations**		
Infrastructural requirements	• Individual units and communal space with senior-friendly fittings, access to basic amenities within proximity. • Land scarcity might affect future infrastructural capacity.	• Designated private room for individuals and common space for socializing. Access to amenities and leisure within proximity. • Land scarcity might affect future infrastructural capacity.
Care quality assessment	Provisions and guidelines are not in policy discussion yet.	
Operational authorization	Development in Progress. The Health Care Services Act 2021 (implemented in three phases from September 2021 till March 2023) will introduce licensure for different long-term care services, including assisted living.	

The analysis above shows that there remain gaps in the meso- and macro-level regulations in the assisted living space in long-term care provision. Below, this paper discusses policy implications of these findings and suggests policy recommendations for countries that are racing against time to address care issues for a gradually rising graying population.

## Discussion

The introduction of ALFs in Singapore is an opportunity to broaden the discussion on the options of long-term care for senior citizens in the future. Despite inheriting a Western influence in its political institutions, Singapore remains very much a collectivist society at its cultural core. In the past few decades, it has espoused a largely Confucianism-influenced filial piety value system in terms of caring for the older generation (22, 23). This care philosophy implies that informal care rendered by family members is perceived as the first line of defense in deciding care options; this is in line with the government's narrative of “family first” (24). This value likely explains why Singapore has been limited in terms of care options in the past, and why the engagement of foreign domestic helpers remain a mainstream choice for many families to date (25, 26). With the changing demographic patterns, informal caregiving is unlikely to be viable for most families in the future. Studies from fast aging countries such as Japan and China have also suggested that depending on informal care only without strengthening formal care provision posed negative social consequences to both the carers and older people and is unlikely to be a tenable long-term approach (27, 28). A study in Singapore showed that many informal caregivers without social and institutional support demonstrated symptoms of depression (29). Furthermore, many older people from the post-war baby boomer generation will likely prefer to exercise more autonomy and self-efficacy in deciding their care arrangements. A recent qualitative study on the baby boomer generation in Singapore reported that this generation tends to value productive aging by pursuing lifelong learning and contributing actively to their families and society, instead of being confined within conventional aging roles (30). In lieu of this shifting preference and changing socio-cultural views on aging, the demand for independent living and assisted living will likely increase in Singapore.

The expansion of both public and private ALFs is a positive measure that would hopefully result in the diversification of long-term care services and the broadening of the long-term care continuum. For a rapidly aging country such as Singapore, the expansion of ALFs is an important mechanism that could help the government to promote aging-in-place and cost savings, while easing the bed crunch in care institutions such as nursing homes. The expansion of assisted living and the strengthening of family support for older people may not be mutually exclusive, as studies conducted in the US have shown that family involvement in assisted living was commonplace and resulted in high satisfaction levels among the residents (31, 32).

As ALFs continue to expand, they will have inevitably to confront a longstanding human resource issue: the shortage of locally trained care workers. Care workers are generally not perceived as desirable jobs due to the inherent stigma and low occupational status associated with the long-term care sector (33, 34). A demanding schedule, limited job prospects, and low pay are other factors that make care work a less appealing career in Singapore (33). Based on a past commissioned study, the long-term care workforce requires an ~130% increase in manpower by 2030 to adequately serve the care needs of older people (34). However, the manpower supply in long-term care has not been increasing proportionately with the demand resulted from an aging population.

Beyond this, there is a need to strengthen processes and institutions governing the assessment of care quality and operations of ALFs. The development of ALFs is just starting in Singapore, and there is currently a lack of a clear monitoring framework and care assessment instrument, factors important for operational audit and authorization. Apart from this, there needs to be a clearer stipulation of the staff–resident ratio for public and private ALFs. While the private ALF has proposed a 1:8 to 1:10 ratio (16), this is unlikely to be tenable in the public ALF model. Furthermore, the limitations on mobilizing night-care workers to effectively monitor the safety of the residents will pose a challenge.

One of the major challenges of expanding assisted living in Singapore is the lack of specificities in its overall governance. While the Health Care Services Act will be implemented in phases from 2021 to 2023, and will ultimately include the regulations for long-term care services, there is currently a lack of explicit soft laws in the form of an interim policy guidelines to address a multitude of issues that would arise in the operations of ALFs, such as land use, fire safety, development planning, and care provisions. Several regulatory questions revolving around issues of planning and development, including increasing the supply-side readiness of the ALFs, require substantial deliberation and planning. For instance, should some commercial land be converted and earmarked for the construction of more long-term care facilities, particularly for ALFs, in the future? How could fire safety and different housing types be designed and planned to serve the different market demands—from lower- and middle-class, to the premium end—for the ALFs? How should different incentives be introduced to boost the supply-side readiness of ALFs in terms of encouraging more private participation or public–private partnerships, and how should novel technologies be deployed in ALFs to enhance operations? These are operationally intricate issues that require an inter-governmental effort and multidisciplinary thinking to prepare for. New regulations need to be piloted to resolve the regulatory ambiguities.

Moving forward, the attitudes toward and preferences for assisted living as a mainstream model of long-term care, plus the willingness to pay for it, will need to be gauged. In addition, quality of life, the depth of social network and social capital of the residents in the ALFs can be accessed early on to determine the effectiveness of assisted living for the older population before they are scaled up nationally in the future. These agendas could be incorporated as the immediate next steps for future research directions. These examinations could be commissioned by either the public agencies or think tanks through population-based surveys and focus group discussions, so that direct insights can be gleaned from the general population to feed back to policy decisions.

Based on Singapore's experience in expanding assisted living as a mainstream long-term care option for a rapidly aging population and the regulatory gaps that have been identified, we offer five recommendations below for countries preparing to chart new territory in long-term care provision (see Table 2 for the summary of policy recommendations).

**Table 2:** Policy recommendations to strengthen the governance of ALFs.

Clear provisions on care quality assessment and redress of grievance for assisted living.
Establish a minimum standard of care for assisted living.
Differential regulations for assisted living.
Routine care assessment for ALF residents to promote dynamism in care provision.
Applying technology in ALFs.

### (i) Set Out Clear Provisions on Care Quality Assessment and the Redress of Grievance for Assisted Living

While a movement to establish good practices and uphold quality assurance exists among private ALF providers in Singapore, and evaluations for the medium-term and long-term assessment of assisted living have been planned by the government, there is a need for regulations to address several areas that are still imbued with ambiguity. For instance, licensure, the stipulation of an acceptable ratio between care staff and residents, the minimum qualifications for various staff members, including care workers and administrative workers, and the governance of liability in ALFs are areas that need more clarity. To this end, there is merit to develop a care quality assessment tool that could enable regulators to audit the providers routinely, as ALFs in North America have done (11). In addition, a public commission or a formal grievance mechanism that allows cases of abuse, neglect, and exploitation of long-term care facilities residents and other grave issues to be highlighted should also be considered. The establishment of an “Elderly Ombudsman Agency” could address cases of aggression and abuse that affect the senior citizens, especially in the long-term care facilities (35).

### (ii) Establish a Minimum Standard of Care for Assisted Living

A minimum standard of care for older people in the long-term care facilities is currently not defined in Singapore. While efforts are underway to establish formal legislations for long-term care, policy guidelines that stipulate the components that constitute a minimum level of care that older people should be provided with are currently lacking in Singapore. Inspiration could potentially be drawn from North American jurisdictions to define the minimum standard of care for ALFs, based on their licensure types. In the US, most states required ALFs to provide certain essential services. ALFs are required to provide 24-h staff, housekeeping, at least two meals a day, assistance with at least two ADLs, and medication administration (36).

### (iii) Develop Differential Regulations for Assisted Living

The proposal to establish different models of ALFs signals that there will be a need for differential regulations for assisted living in the future. A study in the US reported that licensure type did affect policies and practices in assisted living (37). In principle, a purpose-built ALF would be regulated differently from an ALF which is modified and retrofitted from an existing building. The range of service provisions could also differ depending on whether an ALF caters to older population with only limited functional decline or an older population with higher care needs.

### (iv) Put in Place a Routine Care Assessment for ALF Residents to Promote Dynamism in Care Provision

There is also a need to establish a routine care assessment for older people after they are admitted to the ALFs. This routine care assessment will be important in planning for and allocating health and long-term care resources, so that they are better targeted and more comprehensive. For instance, the Japanese government has promoted the use of the “Kihon” checklist as a tool to screen for frailty and to identify older people with a high risk of disability, as well as to align their needs to various prevention programmes in each community to strengthen community-level care and delay institutionalisations (38). It is important to note that the long-term care trajectory for some older adults may not resemble a linear pathway, with their health trajectories more gradual at certain junctures and sharper at other intervals (39). A routine care assessment for assisted living residents should therefore promote more dynamism in care provision by establishing the best form of care that should be rendered to residents in long-term care facilities, including assisted living, at different junctures.

### (v) Apply Technology in ALFs

In view of the shortage of care workers, which is exacerbated by the ongoing Covid-19 pandemic, the long-term care industry, including assisted living, will benefit from the implementation of novel technologies to facilitate operations in the facilities, care surveillance, and service delivery (40). In recent years, robotics and autonomous systems that use social care robots, robotic pets, robotic coaches, and smart home surveillance system have gradually been adopted to assist care workers and caregivers to enhance the care process at home, in the community, and in the institutions (41, 42). However, there are technological risks and ethical issues associated with their deployment which include safety, data security and privacy, liability, autonomy, social connectedness, infantilisation, deception and social justice issues (42). Regulating these novel technologies should warrant as much attention as regulating new models of long-term care. While these cutting-edge technologies are not able to replace care workers, they can be complementary tools deployed to free up time spent on manual tasks, so that they can deliver more personalized care to the residents.

Despite the nascent development of assisted living in Singapore, regulations are taking shape. While certain provisions, such as operational management, monitoring framework, and stipulations of training requirements for staff, need more clarity, overall, most of the regulatory components at the micro-, meso-, and macro-levels are progressing. This paper fills a significant knowledge gap in terms of the governance and regulations of assisted living in the long-term care literature. Forecasting the care needs for the population is crucial as most countries across the world will have to confront the issue of aging populations in the years or decades to come. The insights gleaned from this case study could provide policy lessons for countries aiming to expand assisted living as a policy response to diversify long-term care options for senior citizens. These lessons will allow countries or cities exploring the expansion of assisted living as a care option to reflect on some of the regulatory challenges involved, and to make short-, medium-, and long-term plans to integrate assisted living into the entire care ecosystem provided for the older populations.

## Data Availability Statement

The original contributions presented in the study are included in the article/supplementary material, further inquiries can be directed to the corresponding author.

## Author Contributions

ST and LP collected and analyzed the data. ST wrote the main manuscript text and prepared the tables and figures. All authors were involved in the conceptualization of idea and research questions for this research and reviewed the manuscript.

## Funding

This study was funded by the Intramural Grant from the Saw Swee Hock School of Public Health, National University of Singapore.

## Conflict of Interest

The authors declare that the research was conducted in the absence of any commercial or financial relationships that could be construed as a potential conflict of interest.

## Publisher's Note

All claims expressed in this article are solely those of the authors and do not necessarily represent those of their affiliated organizations, or those of the publisher, the editors and the reviewers. Any product that may be evaluated in this article, or claim that may be made by its manufacturer, is not guaranteed or endorsed by the publisher.
